# The Socio-Communicative Development of Preterm Infants Is Resistant to the Negative Effects of Parity on Maternal Responsiveness

**DOI:** 10.3389/fpsyg.2018.00043

**Published:** 2018-02-02

**Authors:** Ivete F. R. Caldas, Marilice F. Garotti, Victor K. M. Shiramizu, Antonio Pereira

**Affiliations:** ^1^Graduate Program in Neuroscience and Cell Biology, Institute of Biological Sciences, Federal University of Pará (UFPA), Belém, Brazil; ^2^Graduate Program for Research and Theory of Behavior, Institute of Human Sciences, Federal University of Pará (UFPA), Belém, Brazil; ^3^Brain Institute, Federal University of Rio Grande do Norte, Natal, Brazil; ^4^Institute of Technology, Federal University of Pará (UFPA), Belém, Brazil

**Keywords:** preterm, maternal responsivity, parity, social-communicative, development

## Abstract

Humans are born completely dependent on adult care for survival. To get the necessary support, newborns rely on socio-communicative abilities which have both innate and learned components. Maternal responsiveness (MR), as a critical aspect of mother-infant interaction, is a robust predictor of the acquisition of socio-communicative abilities. However, maternal responsiveness (MR) is influenced by parity, since mothers rely on a limited capacity of cognitive control for efficient attachment with their offspring. This fact is of particular concern for preterms, whose developing brain already faces many challenges due to their premature emergence from the womb's controlled environment and may still have to compete with siblings for mother's attention. Thus, in the present work, we aimed to understand how parity interferes with MR and whether it affects the development of socio-communicative abilities of preterm infants. We used the Social Interaction Rating Scale (SIRS) and the mother-child observation protocol in 18 dyads with gestational age <36 weeks. Dyads were separated into three groups: primiparous with twin pregnancy (TPM), primiparous (PM), and multiparous (MP). Dyadic behavior was evaluated at 3, 6, 9, and 12 months. Our results show that offspring size affects MR, but not the socio-communicative development of preterm infants during the first year, suggesting a level of resilience of brain systems supporting the attachment to caregivers.

## Introduction

Preterm birth, characterized by delivery before 37 weeks of gestational age, is on the rise worldwide (Goldenberg et al., 2008; Abbott, 2015). The World Health Organization (WHO) proposes the following sub-categories for preterms, based on gestational age at birth: extremely preterm (< 28 weeks), very preterm (28 to < 32 weeks), and moderate to late preterm (32 to <37 weeks) (Walle et al., 2017). Due to advances in neonatal care, many infants are now able to survive premature birth. However, they face increased risks of neurodevelopmental sequelae (Abbott, 2015) including cognitive and socio-communicative impairments (Saigal and Doyle, 2008; Rogers et al., 2012; Montagna and Nosarti, 2016).

Parenting practices which promote parent-to-infant attachment are a critical modulator of both the course and outcome of child development (Ainsworth and Bowlby, 1991; Meins et al., 2017). Variations in quality of attachment are associated with personality differences later in life (Sroufe, 2005). A key component of attachment is maternal responsiveness (MR), or the mother's ability to detect and respond to the infant's behavioral signals during dyadic interactions (Landry et al., 2001; Feldman et al., 2014). Parents and infants possess both a perceptual and behavioral predisposition to engage in interactions that work to promote attachment (Murray et al., 2016). Some characteristics of infants with clinical conditions, such as cleft lip (Murray et al., 2008), can present challenges for parent-infant interactions (De Pascalis et al., 2017) and compromise attachment. Maternal interactions with preterm infants are also less effective than interactions with infants born at term (Crnic et al., 1983; Harrison and Magill-Evans, 1996). One reason is the decreased ability of preterm infants regarding attention control and facial expressivity during interactions (Bozzette, 2007). Another reason is that premature birth is both a stressful and emotionally demanding experience for parents (Singer et al., 1999; Forcada-Guex et al., 2011; Holditch-Davis et al., 2015; Horwitz et al., 2015; Ionio et al., 2016, 2017). Some preterms need to remain hospitalized in the neonatal intensive care unit (NICU) soon after birth and are kept separate from parents who are thus unable to take care of them for days or even weeks. Such prolonged periods of NICU hospitalization are associated with higher rates of postpartum depression (PPD) in preterm mothers (Tahirkheli et al., 2014; Vasa et al., 2014) and a powerful threat to parent-child attachment. Preterm parents are also susceptible to the Vulnerable Child Syndrome (VCS), whereby children who were at one point in their lives at risk of death continue to be perceived as being more vulnerable, resulting in heightened anxiety to the parents (Green and Solnit, 1964; Horwitz et al., 2015).

Since MR depends on cognitive resources with a limited capacity, close attachments can optimally be formed one person at a time (Klaus and Kennell, 1976). Multiple births are important contributors to the preterm population and also leave parents overloaded with physical, emotional, and financial stresses that contribute to the higher incidence of PPD seen in multiple birth families (Bryan, 2003). Siblings of twins are also more probable to have behavior problems, learning difficulties and language delays (Bryan, 2003). This challenge to MR can be generalized to other contexts associated with the reproductive experience, or parity, of the mother (Jacobs and Moss, 1976; Fish and Stifter, 1993), with an extensive body of literature showing that birth order has an effect on offspring development (Lehmann et al., 2016). For instance, later-born children score lower on cognitive tests than their younger siblings (Black et al., 2016; Lehmann et al., 2016) and also display a less favorable profile regarding personality traits (Black et al., 2016).

The stakes for infants during interactions with a caregiver are very high, and the infant will strive to bond with the caregiver even when this exchange is far from ideal (Coan, 2008). Severe breakdown of attachment is a catastrophic event, leading to delays in brain development, increased risk for psychological disorders, and stunted physical growth (Center on the Developing Child at Harvard University, 2012; Perry and Sullivan, 2014). Fortunately, infants develop adaptive behavioral strategies to deal with eventual breaches in the interaction with the caregiver (Morton, 2016; Provenzi et al., 2017). In the present work, we studied how parity affects MR and, in turn, the socio-communicative development of preterm infants during the first year of life. Even though prematurity is associated with increased developmental risk for the brain, we hypothesize that preterms are able to cope with the challenges imposed by other siblings competing for parental attention and thus buffer eventual negative effects on MR. We charted and compared the evolution of mother-infant interaction of preterms from three experimental groups, differing in the number of siblings, through the first year using two behavioral observation paradigms: The Social Interaction Rating Scale (SIRS) and the Mother-Child Interaction Protocol.

## Materials and methods

### Participants

Preterm infants born during a 4-month period (August–November 2012) who were <37 weeks of gestational age and hospitalized at the neonatal intensive care unit (NICU) of a private hospital in Belém (PA), BRAZIL, were considered for inclusion in this longitudinal, prospective, follow-up study. The study was ethically approved by the Federal University of Pará Ethics Committee (#176.898) and written informed parental consent was obtained on behalf of all participants. Exclusion criteria were the presence of congenital malformations, genetic syndromes detected in the neonatal period, mothers diagnosed with psychiatric conditions, place of residence outside the city of Belém, and newborns transferred from other institutions. The selected dyads were engaged experimentally on five different occasions. On the first meeting, the researchers explained to the mothers the goal and broad outlines of the study. After signature of the informed consent, a second meeting was scheduled for the gathering of sociodemographic and clinical information and the organization of a chronogram for the quarterly experimental sessions with the dyads. About 1 week before the agreed dates, a researcher contacted the mothers and oriented them to feed their infant at least 1 h before the experimental session and to organize the infant's sleeping schedule to maximize the chances he/she would be awake during evaluations.

We managed to recruit 20 infants to participate in the study. One was excluded for missing more than one experimental session, and the other died. The remaining sample was composed of 18 infants and their respective mothers (dyads). The 18 dyads were separated into three groups with the following characteristics: primiparous (PM) (*N* = 6), multiparous (MP) (*N* = 6), primiparous with twins (TPM) (*N* = 6).

### Experimental procedures

Prenatal, perinatal, and sociodemographic data were collected during the hospitalization period (see Table 1). Follow-up visits occurred when the infants were 3, 6, 9, and 12 months old, or, in corrected age (CA), 1, 4, 7, and 10 months old, respectively. Semi-structured sessions took place in a private hospital's room (5.0 × 5.0 m) during daytime and were video recorded with a digital camera (Sony HD SR-45). For each session, a different set of toys was selected according to the age of the infant for free interaction with the mother. Each session lasted for 30 min, divided into three periods of 10 min each. In the first period, the dyad was welcomed by a researcher, and the mother was interviewed about any intervening medical occurrence since the previous session. In the second 10-min period, the mothers were asked to interact freely with the infants trying to help them play. In the last period, the experimenter interacted with the infants for further developmental evaluation. However, for the analysis, we considered only the mother-infant interaction period.

**Table 1:** Sociodemographic characteristics of families and clinical aspects of dyads.

**Variables**	**Total Sample** ***N*****^a^** = **18**		**TPM *n* = 6^b^^,^^c^**		**PM** ***n*** = **6^b^**		**MP** ***n*** = **6^b^**		*p*-value^*^
	**n**	**%**	**n**	**%**	**n**	**%**	**n**	**%**	
**MATERNAL AGE**									
≤ 34 y.o.	12	66.66	4	66.66	6	100.00	2	33.34	0.421
> 34 y.o.	6	33.34	2	33.34	0	0.00	4	66.66	
**MATERNAL EDUCATION**									
Less than middle-school	1	5.55	0	0.00	0	0.00	1	16.66	0.150
Middle-school	17	94.45	6	100.00	6	100.00	5	83.34	
**FAMILY INCOME**									
>1 minimum wage	0	0.00	0	0.00	0	0.00	0	0.00	0.823
1 to 4 minimum wages	4	22.22	2	33.34	1	16.66	1	16.66	
< 5 minimum wages	14	77.78	4	66.66	5	83.34	5	83.34	
**GESTATIONAL AGE**									
28 to < 32 weeks	15	83.34	6	100.00	5	83.34	4	66.66	0.213
32 to < 37 weeks	3	16.66	0	0.00	1	16.66	2	33.34	
**BIRTH WEIGHT**									
≤ 1,500 grams	2	11.11	0	0.00	0	0.00	2	33.34	0.196
1,500 to < 2,500	16	88.89	6	100.00	6	100.00	4	66.66	
> 2,500 grams	0	0.00	0	0.00	0	0.00	0	0.00	
**SEX**									
Male	7	38.88	2	33.34	3	50.00	2	33.34	0.183
Female	11	61.12	4	66.66	3	50.00	4	66.66	
**NICU**									
≤ 10 days	1	5.55	0	0.00	0	0.00	1	16.66	0.691
10 to 20 days	10	55.55	4	66.66	4	66.66	2	33.34	
> 21 days	7	38.90	2	33.34	2	33.34	3	50.00	
**APGAR5**									
≤ 7 points	2	11.11	1	16.66	1	16.66	0	0.00	0.194
> 7 points	16	88.89	5	83.34	5	83.34	6	100.00	
**OXYGEN THERAPY**									
≤ 10 days or less	14	77.77	5	83.34	5	83.34	4	66.66	0.539
> 10 days or greater	4	22.23	1	16.66	1	16.66	2	33.34	
**NEONATAL JAUNDICE**									
Yes	15	83.34	6	100.00	4	66.66	5	83.34	0.290
No	3	16.66	0	0.00	2	33.34	1	16.66	
**INFECTIOUS DISEASE DURING PREGNANCY**^d^									
Yes	14	77.77	5	83.34	4	66.66	6	100.00	0.333
No	4	22.23	1	16.66	2	33.34	0	0.00	

a total number of the sample;

b number of participants per group;

c this group consisted of three mothers of twins;

d *syphilis, toxoplasmosis, rubella, cytomegalovirus and Herpes; TPM (preterm twins of primiparous mothers), PM (preterms of primiparous) and MP (multiparous)*.

* *Used Fisher-Freeman-Halton Test (p < 0.01)*.

The six final minutes of the mother-infant interaction were transcribed and quantitatively analyzed with the help of the software Transana 2.53 (www.transana.org) by two expert judges. The interjudge agreement (I) for each category was based on a randomly-chosen 35% segment length of each session's video recording, according to the following formula: I = [A/(A+D)]x100, where A is the amount of agreements, D the amount of disagreements. The final I was 83% for both the SIRS and the mother-child observation protocol (see below).

### Evaluation instruments

#### Clinical records

Maternal, gestational and obstetric data (complications in childbirth, number of pregnancies, type of delivery, prenatal care, etc.); neonatal (birth weight, gestational age, Apgar, sex, etc.) and postnatal information (need for ventilator support, use of vasoactive drugs, phototherapy, presence of neonatal complications, etc.) (see Table 1).

#### Sociodemographic interview

Composed of data on individual characteristics of the mother and spouse (marital status, religion, etc.), family composition and demographics (family type, number of children and birth order, number of people residing in the household, number of rooms, etc.), data on schooling, income and paid occupation of the parents. This interview was based on adaptations to the script of the Integrated Nucleus of Studies and Research in Development Disorders (NIEPED) and the Brazilian Economic Classification Criterion (CCEB) (www.abep.org) (see Table 1).

#### Social interaction rating scale (SIRS)

The SIRS evaluates the responsiveness of the caregiver/mother in six dimensions: (a) Contingency (mother's response to the child's initiation toward objects and/or events); (b) Directiveness (mother commands and/or directs the attention of the child); (c) Initiation (mother initiates interaction with the child); (d) Level of affection (affective responsiveness, such as praise, encouragement, attention to the child and enthusiasm during parent-child interactions); (e) Level of Movement/Participation (mother engages in physical contact with the child, encouraging participation in activities); (f) Maintenance of the interaction (mother initiates and/or helps the child with the functional use of objects) (Ruble et al., 2008).

Each SIRS dimension was scored according to a 5-point, Likert scale ranging from 1 to 3, with 0.5 midpoints (1, 1.5, 2, 2.5, 3). The score for the six dimensions was summed to yield a general score for each subject (Ruble et al., 2008).

#### Mother-child observation protocol

The infant's engagement with people and objects was evaluated according to the protocol proposed by Bakeman and Adamson (1984) which rates how infants coordinate their attention to people (EP) and objects (EO), individually, or together (EOP). The EP rating included the following behaviors, seeking physical contact, moving the body, looking, smiling, crying and vocalizing toward the caregiver, touching the caregiver, crying and imitating the caregiver. The EO rating included trying to pick up objects, grab objects, move the body toward objects, look and smile toward objects. The EOP rating included pointing or attempting to point to an object, give or show objects, initiate triadic interaction and requesting behaviors.

### Data analyses

For statistical analysis, we used generalized estimating equations (GEE), which are widely used in longitudinal studies when data do not fit a normal distribution and are not homogeneous (variance equality). Since the GEE's standard covariance estimator might inflate type I errors when the sample size is small (Teerenstra et al., 2010), our analysis took into account the estimation of a robust covariance matrix, as suggested by Morel et al. (2003). The Sidak test was used for multiple comparisons. Maternal and infant demographic characteristics and birth outcomes were described by frequencies and percentages (Table 1). Sample characteristics were compared among groups using the Fisher-Freeman-Halton test. The significance level was set at 0.01.

## Results

Table 1 shows the socio-demographic and perinatal characteristics of the dyads. Overall, there was no difference among the groups regarding those variables. Except for the MP group, most mothers were < 34 y.o. The majority had at least a middle-school degree, and the family income was more than 5 Brazilian minimum wages. The majority of infants spent between 10 and 20 days in the NICU and most mothers reported the occurrence of at least one infectious disease during their pregnancy.

Table 2 shows the average SIRS score for each group. The score for the PM group was highest for the ages of six and nine months, respectively, in the following dimensions: contingency (3.00 ± 0.23 and 3.00 ± 0.38), initiation (3.00 ± 0.25 and 3.00 ± 0.60), and movement/participation (3.00 ± 0.25 and 3.00 ± 0.60). The MP group, on the other hand, had its lowest scores at three months of age in the dimension movement/participation (1.50 ± 0.00) while the TPM group had the lowest scores with contingency (1.62 ± 0.21) at 12 months.

**Table 2:** Average Social Interaction Rating Scale (SIRS) scores for each group.

	**Affect**							
	**3 months**		**6 months**		**9 months**		**12 months**	
	**Mean**	**SD**	**Mean**	**SD**	**Mean**	**SD**	**Mean**	**SD**
TPM	2.50	0.47	2.50	0.62	1.75	0.67	1.87	0.54
PM	2.58	0.34	2.75	0.25	2.75	0.38	2.91	0.18
MP	1.75	0.25	2.25	0.25	2.16	0.37	2.08	0.44
**MAINTENANCE**								
TPM	2.25	0.49	2.50	0.58	2.00	0.70	1.87	0.21
PM	2.25	0.38	2.58	0.34	2.66	0.47	2.83	0.23
MP	1.58	0.18	2.41	0.34	2.08	0.34	2.00	0.04
**DIRECTIVENESS**								
TPM	2.00	0.25	2.50	0.25	2.25	0.49	2.00	0.25
PM	2.50	0.25	2.50	0.25	2.75	0.49	2.75	0.27
MP	2.00	0.00	2.25	0.25	1.83	0.47	1.91	0.60
**CONTINGENCY**								
TPM	2.50	0.49	2.50	0.25	2.00	0.66	1.62	0.21
PM	2.50	0.37	3.00	0.23	3.00	0.38	2.91	0.18
MP	1.75	0.27	2.25	0.52	2.25	0.40	2.08	0.44
**INITIATION**								
TPM	2.25	0.38	2.50	0.50	2.00	0.73	1.75	0.25
PM	2.50	0.40	3.00	0.25	3.00	0.60	2.75	0.27
MP	1.58	0.18	2.33	0.47	2.25	0.25	1.91	0.44
**MOVEMENT / PARTICIPATION**								
TPM	2.25	0.40	2.50	0.44	2.00	0.58	1.75	0.28
PM	2.50	0.41	3.00	0.25	3.00	0.60	2.75	0.27
MP	1.50	0.00	2.25	0.38	2.16	0.37	2.00	0.50

*TPM (preterm twins of primiparous mothers), PM (preterms of primiparous) and MP (multiparous)*.

**Figure 1:**
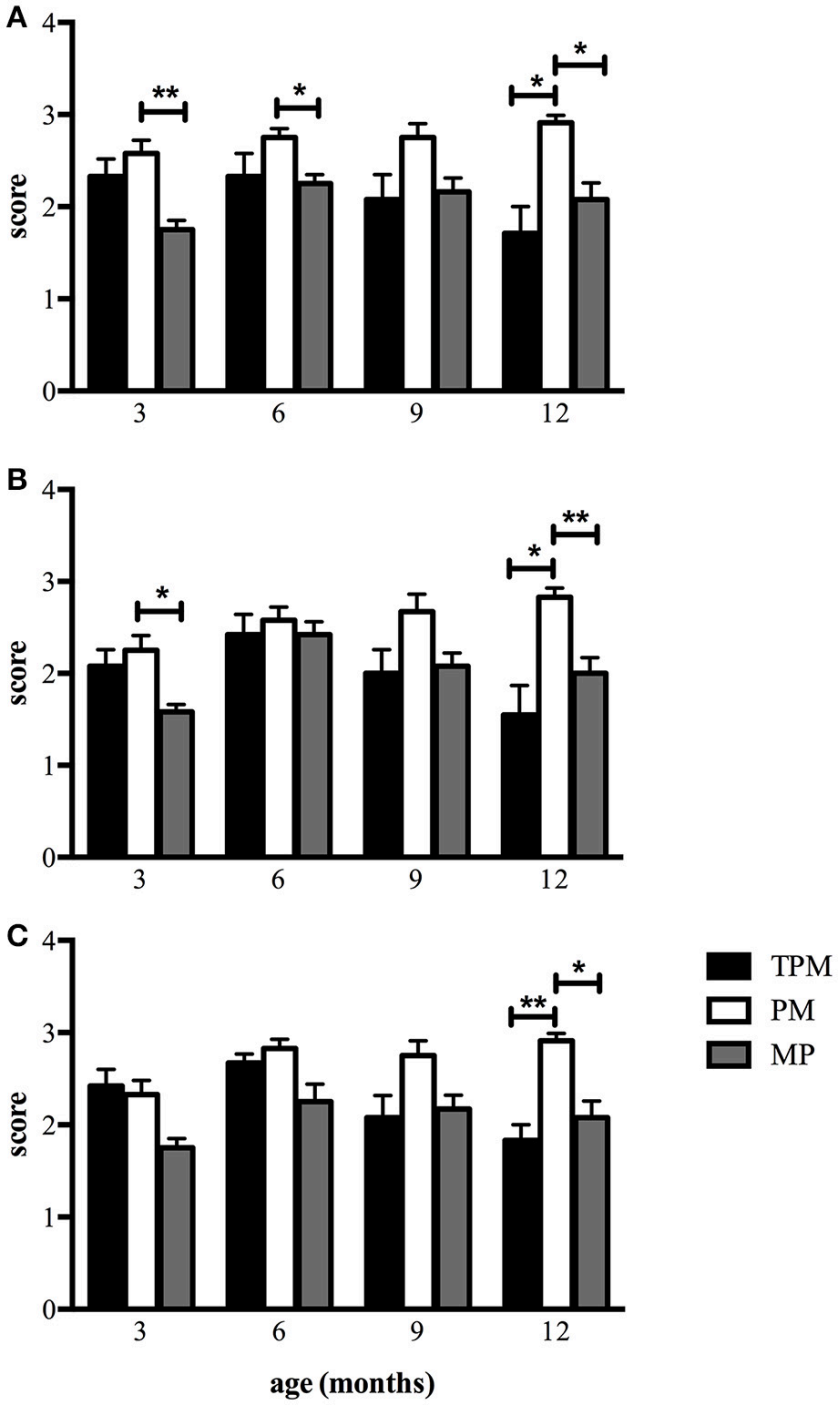
Average scores for the dimensions **(A)** *level of affect*, **(B)** *maintenance*, and **(C)** *contingency* of the *Social Interaction Rating Scale* (SIRS) of mothers from PM, MP, and TPM dyads during the first year of life. Sidak Test (^**^p < *0.0001*, ^*^p < *0.001*).

The GEE revealed interaction effects between *group* and the infants' age for *level of affect* (Wald χ^2^ = 53.12, *df* = 6, *p* < 0.001), *maintenance* (Wald χ^2^ = 34.23, *df* = 6, *p* < 0.001), and *contingency* (Wald χ^2^ = 37.53, *df* = 6, *p* < 0.001). The PM group had higher scores for *level of affect* than the MP group at both 3 (2.58 ± 0.14 vs. 1.75 ± 0.10, *p* < 0.001) and 6 months (2.75 ± 0.10 vs. 2.25 ± 0.10, *p* = 0.035), and also higher scores than both the MP (2.91 ± 0.08 vs. 2.08 ± 0.18, *p* = 0.002) and the TPM (2.91 ± 0.08 vs. 1.71 ± 0.29, *p* = 0.004) groups at 12 months (Figure 1A). The PM group had higher *maintenance* scores than the MP group at both 3 (2.25 ± 0.61 vs. 1.58 ± 0.08 points, *p* = 0.008) and 12 months (2.83 ± 0.10 vs. 2.00 ± 0.17 points, *p* < 0.001), and also higher scores than the TPM group at 12 months (2.83 ± 0.10 vs. 1.55 ± 0.33 points, *p* = 0.008) (Figure 1B). At 12 months, the PM group had *contingency* scores larger than both the MP (2.91 ± 0.08 vs. 2.08 ± 0.18 points, *p* < 0.002) and TPM groups 12 meses (2.91 ± 0.08 vs. 1.83 ± 0.17 points, *p* < 0.001) (Figure 1C).

**Figure 2:**
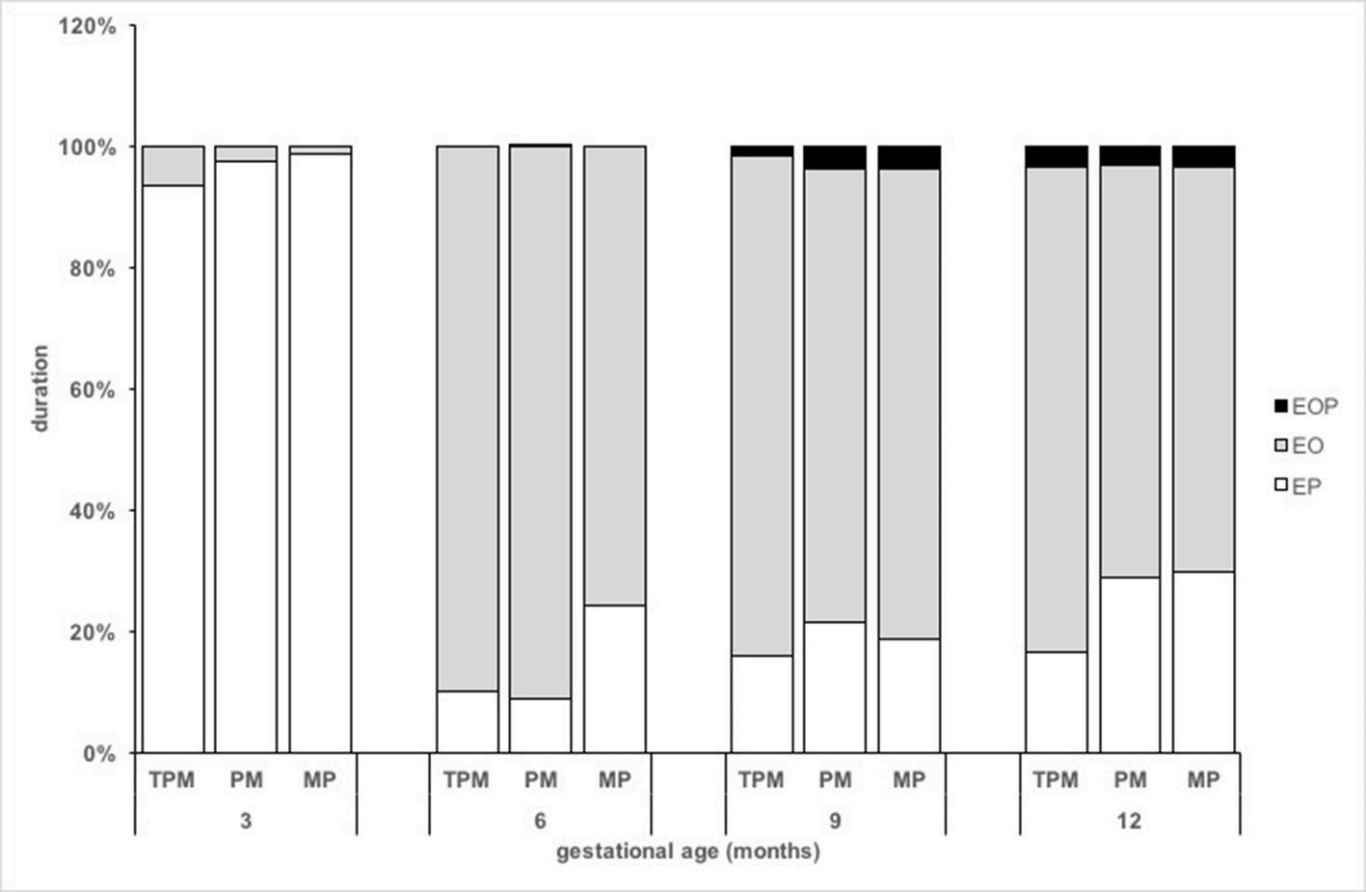
Duration of engagement (in percentage) with (A) person (EP), (B) objects (EO), and (C) person and objects (EOP) of preterms from PM, MP, and TPM dyads during the first year of life.

The results also revealed interaction effects between the infants' age and MR for EP (Wald χ^2^ = 247.99, *df* = 3, *p* < 0.001), EO (Wald χ^2^ = 300.20, *df* = 3, *p* < 0.001), and EOP (Wald χ^2^ = 34.87, *df* = 3, *p* < 0.001). There was no interaction effect between groups. The EP scores were higher at 3 months than at 6 (95.93 ± 14.65 vs. 29.29 ± 15.98, *p* < 0.001), 9 (95.93 ± 14.65 vs. 29.41 ± 18.00, *p* < 0.001), and 12 months (95.93 ± 14.65 vs. 25.41 ± 17.31, *p* < 0.001) (Figure 2). There were significant main effects for the variables contingency (Wald χ^2^ = 5.068, *df* = 1, *p* = 0.024), initiation (Wald χ^2^ = 23.946, *df* = 1, *p* < 0.001), and movement/participation (Wald χ^2^ = 11.211, *df* = 1, *p* < 0.001) on EP. The EO scores were lower at 3 months than at 6 (3.15 ± 15.94 vs. 72.97 ± 19.30, *p* < 0.001), 9 (3.15 ± 15.94 vs. 67.68 ± 18.00, *p* < 0.001), and 12 months (3.15 ± 15.94 vs. 67.16 ± 17.74, *p* < 0.001) (Figure 2). There were significant main effects for the variables initiation (Wald χ^2^ = 27.27, *df* = 1, *p* < 0.001) and movement/participation (Wald χ^2^ = 16.77, *df* = 1, *p* < 0.001) on EO. Finally, for EOP, the scores at 9 months were higher than 3 months (3.07 ± 1.56 vs. −0.17 ± 1.41, *p* = 0.01), and at 12 months were higher than 3 (3.45 ± 1.56 vs. −0.17 ± 1.41, *p* = 0.005) and 6 months (3.45 ± 1.69 vs. 0.13 ± 1.09, *p* = 0.027) (Figure 2). There were significant main effects for the variables initiation (Wald χ^2^ = 9.645, *df* = 1, *p* = 0.002) and movement/participation (Wald χ^2^ = 8.262, *df* = 1, *p* = 0.004) on EOP.

## Discussion

Humans are slow-growing and have an extended period of juvenile dependence which demands a considerable amount of parenting investment. Females provide most of the investment to offspring in the majority of mammalian species and display high sensitivity and responsiveness to her infant's signals and communications. Parenting practices, including MR, depend in great extent of cognitive control, subserved by executive functions with a limited capacity (Crandall et al., 2015). Cognitive control can be decreased due to executive dysfunction associated with maternal attention deficit hyperactive disorder (ADHD) or due to stressful situations (Crandall et al., 2015; Sturge-Apple et al., 2017). The presence of multiple young children at the same time is a source of stress due to limited resources and also a significant challenge to cognitive control abilities (Salmon, 2005). Thus, we propose that our present results showing that primiparous mothers have better MR scores than multiparous mothers are due in part to the adverse effect of the number of offspring on cognitive control abilities. A previous study had already shown that deficits in executive functioning are associated with poor MR (Chico et al., 2014). Another study showed that twin births also have a negative impact on MR (Beer et al., 2013).

In the present study, the maternal cognitive control an MR was also probably affected by the stressful situations associated with preterm birth. All subjects of our sample spent at least 10 days at the NICU. As shown before, NICU hospitalization has a large emotional impact on parents (Obeidat et al., 2009) which can last longer due to VCS (Green and Solnit, 1964; Horwitz et al., 2015).

Surprisingly, our results also show that the negative effects of reproductive experience on MR did not seem to have an impact on the socio-communicative abilities of preterm infants. Preterm birth is a leading risk factor for neurobehavioral development (Aarnoudse-Moens et al., 2009). However, not much is known about its specific impact on the development of socio-communicative skills (Zmyj et al., 2017). In theory, preterm birth should contribute to make infants more dependent on parental attention due to its negative effect on the development of executive functions. Thus, the quality of MR should display a strong positive correlation with developmental outcomes regarding socio-communicative abilities. Our results, however, challenge this assumption and show that the development of socio-communicative abilities of preterm infants is not affected by the negative effects of parity on MR. Earlier studies had already shown that infants attach to their caregiver regardless of the quality of care they receive (Glaser, 2000; Sullivan, 2012). Though this behavior comes at a high cost in terms of increased risk for future psychopathologies (Glaser, 2000; Bick, 2012), it is a striking example of self-preservation at such vulnerable phase of human development. A secure attachment can protect or buffer the developing brain from the deleterious effects of increased cortisol levels, for instance (Gunnar, 1998). Future research should further investigate the implications and neural mechanisms associated with this adaptive behavioral flexibility in preterms.

The present study is the first to investigate the effect of reproductive experience on MR and the social-communicative development of preterm infants during their first year of life. Our results contribute to the general understanding of how the social environment, particularly mother-child interactions, can influence the development of preterm infants. This knowledge is essential since delayed childbearing (Johnson et al., 2012) and the rise of *in vitro* fertilization (IVF) (Kamphuis et al., 2014) have contributed to increasing the rate of preterm and multiple births (Homrich da Silva et al., 2008). Since multiple birth and prematurity are interlinked, this phenomenon is of great importance in terms of public health and requires a comprehensive set of prenatal and postnatal measures to guarantee the best outcomes for the affected families. There is a need for specific interventions aimed at preterm infants to help them overcome their developmental odds. Given the role of lower socioeconomic status as an important stressful factor in multiple-birth families, it is advisable to implement specific measures to help low-income households in this situation.

One limitation of our study is the relatively small sample size and the absence of comparison with matched term neonates. Future studies should compensate for this shortcoming and also include subjects from different cultural and socioeconomic backgrounds. Another limitation of the study is the possibility of a Hawthorne effect (McCambridge et al., 2014). Although mothers were asked to play with their child as they usually would, we cannot eliminate the possibility of them modifying their behavior as a result of being video recorded.

## Author contributions

IC, MG, and AP designed the experiments; IC and MG collected data; IC, MG, VS, and AP analyzed data; IC and AP wrote the manuscript.

### Conflict of interest statement

The authors declare that the research was conducted in the absence of any commercial or financial relationships that could be construed as a potential conflict of interest.
